# Preservation of eGFRcre for 1 year with HIF-PHI in non-dialysis patients: a retrospective observational cohort study

**DOI:** 10.1186/s40780-025-00527-1

**Published:** 2025-12-20

**Authors:** Tomohiro Aigami, Tomoyuki Ishigo, Mai Miyao, Masatoshi Nonoyama, Tomohisa Yamashita, Masayuki Koyama, Satoshi Fujii, Toshiyuki Yano, Masato Furuhashi, Masahide Fukudo, Takaki Toda

**Affiliations:** 1 Department of Pharmacy, Sapporo Medical University Hospital, South 1, West 16, Chuo-Ku, Sapporo, 060-8543 Japan; 2 Department of Nephrology and Dialysis Therapy, Sapporo Central Hospital, 1-50, South 9, West 10, Chuo-ku, Sapporo, 064-0809 Japan; 3 Department of Cardiovascular, Renal, and Metabolic Medicine, Sapporo Medical University School of Medicine, South 1, West 16, Chuo-ku, Sapporo, 060-8543 Japan; 4 Department of Public Health, Sapporo Medical University School of Medicine, South 1, West 16, Chuo-Ku, Sapporo, 060-8543 Japan; 5 Department of Clinical Pharmacology, Faculty of Pharmaceutical Sciences, Hokkaido University of Science, 4-1, 15 Maeda 7, Teine-ku, Sapporo, 006-8585 Japan

**Keywords:** HIF-PHI, ESA, eGFRcre, Non-dialysis

## Abstract

**Background:**

Hypoxia-inducible factor prolyl hydroxylase inhibitors (HIF-PHIs) effectively increase hemoglobin levels to treat anemia in patients with chronic kidney disease (CKD). However, there is a paucity of studies on the effect of HIF-PHIs on renal outcomes, such as estimated glomerular filtration rate based on serum creatinine (eGFRcre). Therefore, this study aimed to examine the trajectory of eGFRcre decline following initiation of treatment with HIF-PHI and erythropoiesis-stimulating agents (ESAs) in non-dialysis patients.

**Methods:**

This single-center, retrospective study enrolled non-dialysis patients who were prescribed HIF-PHIs between January 1, 2020 and December 31, 2022. We used five HIF-PHIs approved in Japan. The eGFRcre slope for a year after HIF-PHIs therapy was initiated was compared with that of ESAs. The eGFRcre slope was calculated using a linear mixed-effects model for repeated measures over a year from the time of enrollment.

**Results:**

This study included 134 patients: 79 and 55 patients treated with HIF-PHIs and ESAs, respectively. The model adjusting for time, drug (HIF-PHI or ESA), their interaction, age, sex, BMI, and CKD stage showed that the effect on eGFRcre differed between HIF-PHIs and ESAs (−2.11 ± 0.57 mL/min/1.73 m^2^ per year vs. −3.27 ± 0.17 mL/min/1.73 m^2^ per year, *p* = 0.046). Subgroup analysis by CKD stage revealed a difference in the eGFRcre slope between HIF-PHI and ESA only in the stage 4 group (−1.89 ± 0.44 mL/min/1.73 m^2^ per year vs. −3.69 ± 0.44 mL/min/1.73 m^2^ per year, *p* < 0.001).

**Conclusions:**

HIF-PHI preserved eGFRcre for a year, suggesting that its effect was superior to that of ESA.

**Supplementary Information:**

The online version contains supplementary material available at 10.1186/s40780-025-00527-1.

## Background

Anemia is a common complication of chronic kidney disease (CKD) that worsens with CKD progression [1]. Anemia in CKD is also linked to an increased risk of progression to end-stage kidney disease [2, 3]. Standard treatment medications for anemia included erythropoiesis-stimulating agents (ESAs) and iron supplementation. Recently, Hypoxia-inducible factor prolyl hydroxylase inhibitors (HIF-PHIs), including roxadustat, vadadustat, daprodustat, enarodustat, and molidustat, have demonstrated greater or non-inferior efficacy than ESAs or placebo in clinical trials [4–13]. Moreover, previous systematic reviews have shown that HIF-PHI management decreases the proportion of patients requiring blood transfusions compared to ESAs or placebo [14]. Consequently, HIF-PHIs have been approved for the treatment of anemia in dialysis and non-dialysis patients with CKD in Japan. Moreover, unlike existing injectable ESA, HIF-PHIs can be administered orally, improving patient adherence and wider acceptance in the future.

The treatment of anemia is associated with retention of the estimated glomerular filtration rate (eGFR) [15]. However, several reports have indicated that the treatment of anemia with ESAs is not associated with improved renal outcomes [16, 17]. Experimental studies have suggested that HIF-PHIs may be beneficial in alleviating hypoxic kidney injury [18, 19]. Furthermore, a clinical trial with enarodustat showed that eGFR was maintained in non-dialysis patients; however, the correlation of this efficacy with the correction of anemia was not discussed [10]. In a phase 3 trial, roxadustat showed greater or equivalent eGFR decline compared with placebo or darbepoetin alfa [20, 21]. However, roxadustat, daprodustat, and vadadustat did not demonstrate superior efficacy compared with darbepoetin alfa in preventing CKD progression [6, 8, 10]. Consequently, a meta-analysis incorporating clinical trials of HIF-PHIs revealed no significant effect on renal outcomes when compared with ESAs and placebo [22]. Thus, there is a paucity of consistent data regarding the effect of HIF-PHIs on renal outcomes in clinical practice. The Kidney Disease: Improving Global Outcomes (KDIGO) guidelines recommend further study in this area [23].

Given the limited data on the effect of HIF-PHIs on renal outcomes, such as eGFR, and recent validation of the eGFR slope as a surrogate renal outcome for progression to end-stage kidney disease (ESKD) [24], we hypothesized that HIF-PHI would preserve eGFR based on serum creatinine (eGFRcre) in non-dialysis patients. Therefore, this study aimed to examine the trajectory of eGFRcre decline following initiation of treatment with HIF-PHI and ESAs in non-dialysis patients.

## Methods

### Study design and patients

This single-center, retrospective cohort study was conducted at Sapporo Medical University. The inclusion criteria included: 1) patients with CKD not requiring renal replacement therapy, who were prescribed a HIF-PHI or ESA for the first time between January 1, 2020 and December 31, 2022; 2) patients aged ≥18 years and with at least three eGFRcre values (including one measurement before and one after initiation of HIF-PHI or ESA therapy). The exclusion criteria were as follows: 1) loss to follow-up within 3 months; 2) history of blood transfusions after initiating HIF-PHI or ESA therapy; 3) patients scheduled to initiate renal replacement therapy (RRT) (including hemodialysis, peritoneal dialysis, continuous renal replacement therapy, and kidney transplantation) at the initiation of HIF-PHI or ESA therapy; and 4) missing data.

The HIF-PHIs used in this study were roxadustat, daprodustat, vadadustat, molidustat, and enarodustat, whereas the ESAs used were darbepoetin alpha and epoetin beta-pegol.

### Outcomes

The primary outcome was the difference in the eGFRcre slope between patients initiated with HIF-PHI therapy and those on ESA therapy. Secondary outcomes were 1) the incidence rate of RRT after initiation of HIF-PHI or ESA, 2) the change in eGFRcre slope before and after initiation of HIF-PHI or ESA therapy, and 3) the differences in hemoglobin trend between the HIF-PHI and ESA treatment groups. Furthermore, we categorized the patients according to CKD stage and examined the difference in eGFRcre slope initiation between HIF-PHI and ESA therapy as a subgroup analysis. This analysis was performed because the effectiveness of HIF-PHIs is dependent on residual renal function and CKD stage. In the observation of the eGFRcre slope and hemoglobin trends, the values before the introduction of RRT were used to analyze cases in which RRT was initiated.

### Data collection

We collected patient data, including age, sex, body weight, height, eGFRcre, primary kidney disease (including diabetic kidney disease, hypertensive nephropathy, and chronic glomerular nephropathy), comorbidities (including diabetes mellitus, hypertension, heart failure, dyslipidemia, and cancer), comedications (including iron supplementation, angiotensin receptor blocker (ARB) or angiotensin-converting enzyme inhibitor (ACE-i), angiotensin receptor neprilysin inhibitor (ARNI), mineralocorticoid receptor antagonist (MRA), loop diuretic, and thiazide diuretic), hemoglobin, plasma ferritin, plasma iron, total iron binding capacity (TIBC), and the date of initiating HIF-PHI or ESA therapy, from the electronic medical records. The eGFRcre and hemoglobin levels were recorded monthly. Transferrin saturation (TSAT) was calculated using plasma iron and TIBC. We also recorded add-on medications (nonsteroidal anti-inflammatory drugs, ARB, ACEI, ARNI, MRA, loop diuretics, sodium-glucose co-transporter 2 inhibitors (SGLT2-i), and iron supplementation) after initiating HIF-PHI or ESA.

### Statistical analysis

Continuous variables are summarized as mean and standard deviation or standard error for normally distributed data and as median (interquartile range) for non-normally distributed data. Categorical variables were summarized as counts and percentages. Appropriate between-group differences were evaluated using the Welch’s or Wilcoxon test for continuous variables and the chi-squared test for categorical variables. In addition, post hoc testing for multiple comparisons was performed using the Tukey‒Kramer method for continuous data. The Spearman’s correlation coefficient was used to test the correlations between two continuous variables. Further, we used the cumulative incidence function to calculate the cumulative incidence of RRT.

The eGFRcre slope was calculated using a linear mixed-effects model for repeated measures. First, we assembled an initial model that included time, drug (HIF-PHI or ESA), and their interaction as fixed effects (model 1) to assess differences in the eGFRcre slope between HIF-PHI and ESA. Subsequently, we extended the model to include potential covariates such as age, sex, BMI, and CKD stage to adjust for their effects on eGFRcre (model 2). When analyzing differences in eGFR slope before and after initiation of HIF-PHI therapy, we incorporated time, a “before and after” category, other aforementioned covariates, and the relationship between the “before and after” category and time as a fixed effect. In all cases, we incorporated the individual and intercept of the line as a random effect. Residual log-likelihood was used to assess the goodness-of-fit of the model. The covariance structure of the errors was adjusted to an autoregressive structure, and the restricted maximum-likelihood method was used for parameter estimation. The eGFRcre slope was calculated using all eGFRcre values during the calculation period (−1 to 1 year). For cases where RRT was initiated, eGFRcre values recorded before RRT initiation were used. These models assume that any missing eGFRcre value could be estimated using the eGFRcre data for other individuals together and other covariates in the model. Further, eGFRcre was calculated using the Japanese eGFR formula: eGFRcre (mL/min/1.73 m^2^) = 194*(serum creatinine)-1.094*(age)-0.287(*0.739 if female) [25]. CKD categories were defined as follows: stage 3b, eGFRcre > 30 and ≤45 mL/min/1.73 m^2^; stage 4, eGFRcre > 15 and ≤30 mL/min/1.73 m^2^; stage 5, eGFRcre ≤15 mL/min/1.73 m^2^ [26].

We used the software program JMP® Pro 17.0 (SAS Institute Inc., Cary, NC, USA) for statistical analysis, and a two-sided p-value of < 0.05 was considered statistically significant.

## Results

### Patient characteristics

**Fig. 1:**
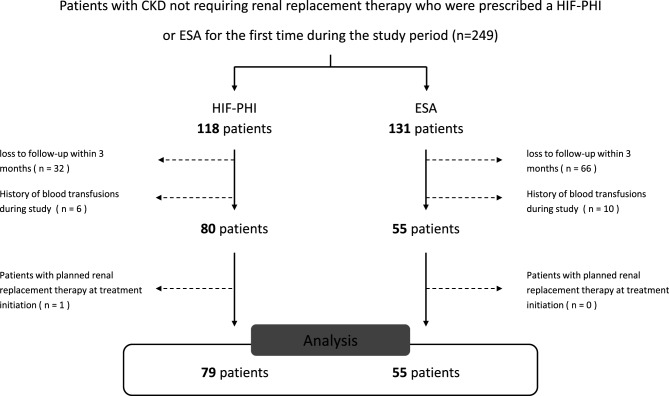
Flow chart of the inclusion criteria for the study participants, abbreviations: CKD, chronic kidney disease; HIF-PHI, hypoxia-inducible factor prolyl hydroxylase inhibitor; ESA, erythropoiesis-stimulating agent

During the study, 134 of 249 patients with CKD who did not require RRT on initiation of treatment with HIF-PHI or ESA were eligible for inclusion (Fig. 1). The median age was 73.5 (interquartile range, 62.5‒80) years, with 45.5% female patients. HIF-PHI therapy was initiated in 59.0% of patients (Table 1). Among those on HIF-PHI therapy, the median eGFRcre was 21.0 (12.9–28.0) mL/min/1.73 m^2^, and the proportions of patients with CKD stages 3b, 4, and 5 were 21.5%, 46.8%, and 31.7%, respectively. The median hemoglobin level at enrollment was 10.0 (9.3‒10.4) g/dL. The proportion of patients on roxadustat, daprodustat, vadadustat, molidustat, and enarodustat were 15.2%, 67.1%, 7.6%, 3.8%, and 6.3%, respectively. (Table S1).

**Table 1:** Baseline characteristics

	ALL *n* = 134	HIF-PHI *n* = 79	ESAs *n* = 55	p-value
Age median (IQR)	73.5 (62.5–80)	73 (60–80)	74 (65–81)	0.614
Female n (%)	61 (45.5)	34 (43.0)	27 (49.1)	0.489
BW median (IQR)	60.85 (47.0–69.2)	62.0 (50.1–69.2)	55.4 (43.0–69.3)	0.088
Height median (IQR)	160.3 (152.3–167.2)	161 (153.5–168.4)	156.8 (151.5–165.5)	0.330
BMI median (IQR)	22.97 (20.37–26.25)	23.72 (21.12–26.69)	22.04 (18.86–25.38)	0.045
BSA median (IQR)	1.61 (1.42–1.79)	1.65 (1.47–1.79)	1.54 (1.27–1.78)	0.075
eGFRcre median (IQR) (mL/min/1.73 m^2^)	21.3 (14.2–28.8)	21.0 (12.9–28.0)	21.7 (15.4–30.8)	0.333
CKD category n (%)				
3b	31 (23.13)	17 (21.52)	14 (25.45)	0.588
4	65 (48.51)	37 (46.84)	28 (50.91)	
5	38 (28.36)	25 (31.65)	13 (23.65)	
Primary kidney disease				
DKD n (%)	39 (29.1)	23 (29.1)	16 (29.1)	0.956
HTN n (%)	36 (26.9)	20 (25.3)	16 (29.1)	
CGN n (%)	24 (17.9)	14 (17.7)	10 (18.2)	
PKD n (%)	9 (6.7)	5 (6.3)	4 (7.3)	
Others n (%)	26 (19.4)	17 (21.5)	9 (16.4)	
Laboratory test				
Hemoglobin median (IQR) (g/dL)	9.8 (8.9–10.3)	10.0 (9.3–10.4)	9.5 (8.6–10.2)	0.013
Ferritin median (IQR) (ng/mL)	173 (82.7–366)	191 (93.7–359)	131.5 (72.6–376.5)	0.141
TSAT median (IQR) (%)	25.2 (18.0–32.0)	26.1 (20.9–32.4)	22.3 (12.6–30.8)	0.057
Albumin median (IQR) (g/dL)	3.5 (3.1–3.8)	3.6 (3.2–3.8)	3.5 (2.9–3.9)	0.652
CRP median (IQR) (mg/dL)	0.13 (0.06–0.48)	0.15 (0.04–0.58)	0.10 (0.10–0.33)	0.998
Concomitant				
Heart failure n (%)	40 (29.9)	16 (20.3)	24 (43.6)	0.004
Hypertension n (%)	103 (76.9)	63 (79.6)	40 (72.7)	0.407
Diabate mellites n (%)	51 (38.1)	29 (36.7)	22 (40.0)	0.721
Dyslipidemia n (%)	75 (56.0)	46 (58.2)	29 (52.7)	0.597
Cancer n (%)	41 (30.6)	27 (34.2)	14 (25.5)	0.342
Comedications				
Iron supplementation n (%)	36 (26.9)	19 (24.1)	17 (30.9)	0.431
ARB/ACE-i n (%)	73 (54.5)	41 (51.9)	32 (58.2)	0.487
ARNI n (%)	13 (9.7)	13 (16.5)	0 (0)	< 0.001
MRA n (%)	26 (19.4)	13 (16.5)	13 (23.6)	0.375
Loop diuretic n (%)	68 (50.8)	38 (48.1)	30 (54.6)	0.487
Thiazide diuretic n (%)	15 (11.2)	9 (11.4)	6 (10.9)	1.000

Values are presented as means ± standard deviation or median (interquartile range) number (with percentage). *p* < 0.05 was considered statistically significant. Abbreviations: BW, Body weight; BMI, Body mass index; CKD, Chronic kidney disease; DKD, Diabetic kidney disease; HTN, Hypertensive nephropathy; CGN, Chronic glomerular nephropathy; PKD, Polycystic kidney disease; TSAT, Transferrin saturation; CRP, C-reactive protein; ARB, angiotensin receptor blocker; ACE-i, angiotensin-converting enzyme inhibits; ARNI, angiotensin receptor neprilysin inhibitor; MRA, mineralocorticoid receptor antagonist

Baseline characteristics differed between the groups. Notably, BMI differed between the drug groups (*p* = 0.045). No differences in CKD category and primary kidney disease were observed between the groups (*p* = 0.588, 0.956). Hemoglobin at time of screening differed between both groups (10.0 (9.3‒10.4) g/dL vs. 9.5 (8.6‒10.2) g/dL, *p* = 0.013); however, no difference was observed in plasma ferritin (191 (93.7‒359) ng/mL vs. 131.5 (72.6‒376.5) ng/mL, *p* = 0.141), TSAT (26.1 (20.9‒32.4) % vs. 22.3 (12.6‒30.8) %, *p* = 0.057), Alb (3.6 (3.2–3.8) g/dL vs. 3.5 (2.9–3.9) g/dL, *p* = 0.652) and CRP (0.15 (0.04–0.58) mg/dL vs. 0.10 (0.10–0.33) mg/dL, *p* = 0.998) between the group. In the coexisting conditions, the proportion of heart failure was lower in the HIF-PHI group than in the ESA group (20.3% vs. 43.6%, *p* = 0.004). Furthermore, no differences in other coexisting conditions were observed between the groups at the time of screening. The percentage of patients on ARNI was higher in the HIF-PHI group (16.5% vs. 0.0%, *p* < 0.001). Other medications did not differ between the groups at the time of screening (Table 1). The add-on medications after initiating HIF-PHI or ESA are shown in Supplemental Information (Table S2).

### RRT incidence

During the study period, 6 (7.6%) patients in the HIF-PHI group and 11 (20.0%) patients in the ESA group started RRT. The cumulative incidence of 1-year RRT after initiating therapy with HIF-PHI and ESA agents during the study period was 9.4% and 24.7%, respectively. This difference was statistically significant (*p* < 0.001; Fig. S1).

### The eGFRcre slope

**Fig. 2:**
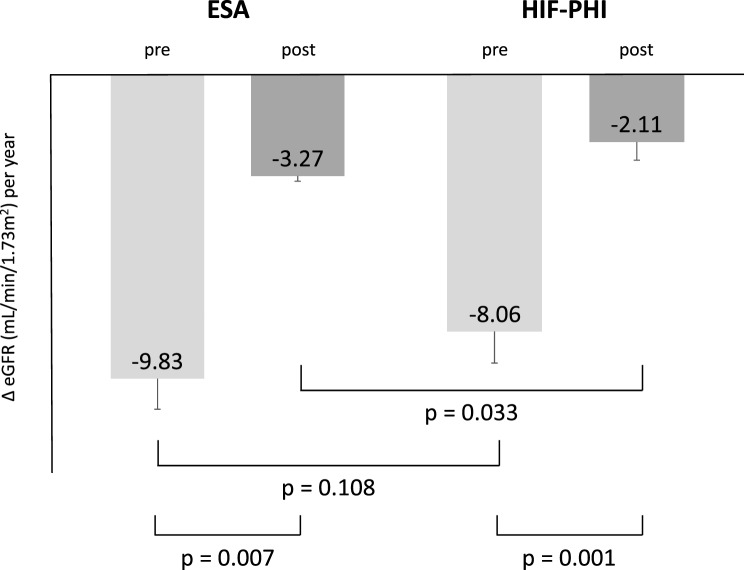
The pre- and post-treatment eGfrcre slope of HIF-PHIs and ESAs, abbreviations: HIF-PHI, hypoxia-inducible factor prolyl hydroxylase inhibitor; ESA, erythropoiesis-stimulating agent

The eGFRcre slope of treatment with HIF-PHI was −2.11± 0.57 mL/min/1.73 m^2^ per year, and that of ESA was−3.27 ± 0.17 mL/min/1.73 m^2^ per year (Fig. 2). Model 1 revealed a significant interaction between time and drug (*p* = 0.033; Fig. 2), indicating that the effect of the drugs on eGFRcre changed over time and differed between the two drug classes (Table 2). Upon including covariates in the extended model (model 2), CKD stage was also a significant factor (*p* < 0.001), in addition to the interaction between time and drug (*p* = 0.046; Table 2).

**Table 2:** Multivariable analysis

	Model 1			Model 2		
Fix effect	Estimate (SE)	95% CI	p-value	Estimate (SE)	95% CI	p-value
Intercept	24.50 (1.21)	22.1 to 26.9	< 0.001	21.98 (3.68)	14.7 to 29.2	< 0.001
Drug (ESA)	−0.081 (0.88)	−1.82 to 1.66	0.927	−0.852 (0.05)	−1.88 to 0.17	0.103
Time month	−0.182 (0.05)	−0.27 to −0.09	< 0.001	−0.176 (0.05)	−0.27 to −0.08	< 0.001
Drug*time	−0.101 (0.05)	−0.19 to −0.01	0.033	−0.097 (0.05)	−0.19 to −0.01	0.046
Age				0.067 (0.04)	−0.01 to 0.14	0.086
Sex (female)				−0.649 (0.52)	−1.69 to 0.39	0.218
BMI				−0.080 (0.11)	−0.29 to 0.13	0.455
CKD stage						
3b				10.95 (0.82)	9.32 to 12.6	< 0.001
4				0.272 (0.69)	−1.09 to 1.63	0.693

*p* < 0.05 was considered statistically significant. Abbreviations: SE, standard error; ESA, erythropoiesis-stimulating agents; BMI, body mass index; CKD, chronic kidney disease

The HIF-PHI and ESA groups showed different eGFRcre slopes before and after treatment (*p* = 0.001 and 0.007, respectively; Fig. 2).

### Hemoglobin trends and the correlations between eGFR change and hemoglobin change

The hemoglobin trends with initiated HIF-PHI and ESA therapy were 1.11 ± 0.09 and 1.17 ± 0.09 g/dL per year, respectively. However, no difference in the hemoglobin trend was observed between the groups (*p* = 0.554; Fig. 2). Before HIF-PHI therapy, a positive correlation was observed between eGFRcre slope and hemoglobin trend (*ρ* = 0.377, *p* = 0.001), whereas non-correlation (*ρ* = 0.017, *p* = 0.882) was observed after initiated HIF-PHI (Fig.S3). No correlations between the eGFRcre slope and hemoglobin trends with ESA were observed both before and after initiation of therapy. No correlation was observed between baseline hemoglobin levels and eGFRcre slope in either group (Fig. S4).

### eGFR slope divided with CKD stage

A significant difference was observed between the eGFRcre slope of therapy with HIF-PHI and that of ESA in patients with stage 4 CKD (−1.89 ± 0.44 mL/min/1.73 m^2^ per year vs.−3.69 ± 0.44 mL/min/1.73 m^2^ per year, *p* < 0.001; Fig.S5). In patients with stages 3b and 5 CKD, no difference in eGFRcre slope was observed (*p* = 0.474, 0.391).

## Discussion

This study revealed that the impact of the drugs on eGFRcre varies not only over time but also between HIF-PHI and ESA, with HIF-PHI being associated with a slower decline in eGFRcre (so-called “eGFRcre slope”) compared with ESA. The model, adjusted for covariate factors, also showed that the effects of HIF-PHI and ESA on eGFRcre differed. However, the differences in the effects of HIF-PHI may vary depending on the CKD stage. To the best of our knowledge, this is the first study to examine the effects of HIF-PHI and ESA on eGFRcre slope in clinical practice.

The effect of HIF-PHIs on renal outcomes remains unclear. However, experimental studies have demonstrated its effects on the protection of renal function in models of acute kidney injury [18, 19]. The Phase 3 clinical trial has demonstrated an eGFR maintenance effect for 24 weeks with enarodustat [10]. However, no similar effect is observed with other HIF-PHIs. Clinical trials of roxadustat and daprodustat reported no significant change in the eGFR slope compared to controls [4, 8]. Molidustat did not appear to affect the eGFR during the study, although it was not statistically significant [12]. A meta-analysis incorporating these trials of HIF-PHI reported no significant effect on renal outcomes when compared with ESAs and placebo [22]. Consequently, further research is required to explore the impact of progressive CKD and the presence of proteinuria/albuminuria on the efficacy of HIF-PHI [23]. The results of this study, which maintained eGFRcre for a year and the association of progression to ESKD with eGFR slope [27], suggest that initiating HIF-PHI may delay the risk of progression to ESKD. Furthermore, the effect was more significant than that of ESAs. In this study, both HIF-PHI and ESA initiation improved hemoglobin levels over time, similar to the results of previous studies [4, 6, 8, 10, 12]. The correlation between the eGFRcre slope and hemoglobin trend changed before and after HIF-PHI initiation but not before and after ESA initiation. The correlation was not observed between baseline hemoglobin levels and eGFRcre slope in either group.　Therefore, although correction of anemia is reportedly associated with the retention of eGFRcre [28], the effect of HIF-PHI on eGFRcre may have additional unique effects. In addition, to minimize the renoprotective effects of ARNI and SGLT-2 inhibitors, the analysis was similar to the primary results after excluding cases in which these agents were added (Table S3). Furthermore, HF and renal function are closely interrelated, and fluctuations in eGFR can occur depending on HF management [29]. Even after adjusting for a history of HF as a covariate in additional analysis, the differences between drug groups remained statistically significant (Table S4). These results support the unique effect of HIF-PHI on eGFRcre.

Although the kidneys are oxygen-consuming organs with high energy demands, they have low oxygen uptake efficiency due to arteriovenous oxygen shunting [30]. Improving the ischemic condition in the kidneys protects against acute kidney injury, which is directly affected, as well as slowing the progression of CKD. Improvement in anemia is predicted to be involved in the improvement of ischemic conditions and the protection of renal function. ESAs for anemia are not associated with renal outcomes, which may be partly due to increased exogenous erythropoietin (EPO) concentrations [16, 17]. On the other hand, HIF-PHI stabilizes HIF-2α to act on EPO-producing cells in the kidneys and liver to promote endogenous EPO production and subsequent hematopoiesis [31]. The different concentrations of endogenous and exogenous EPO may be related to differences in their effects on eGFRcre. Given the composite nature of several mechanisms by which HIF-PHI affects renal function as follows: 1) increased renal blood flow by promoting angiogenesis via VEGF; 2) inhibition of renal cell fibrosis; 3) reduction of oxidative stress by promoting antioxidant expression; 4) anti-inflammatory effects by promoting anti-inflammatory cytokine production; and 5) stabilization of energy metabolism mechanisms, such as mitochondrial dynamics [30–33]. In particular, the inhibition of fibrotic processes and promotion of VEGF-mediated angiogenesis have been shown by stabilization of HIF by PHD inhibition [34], and these effects may explain why HIF-PHI is more renoprotective than ESA.

In this study, the effect of HIF-PHI on eGFRcre differed according to the CKD stage. We attribute this effect to differences in the degree of renal function and pathophysiology at each stage. In stage 3b, there was more residual renal function than in other stages, which may promote EPO production and improve oxygenation more effectively. Therefore, there may be no significant differences in the effects of HIF-PHIs and ESA. However, in stages 4 and 5, the kidneys have little compensatory capacity, and while increased EPO production by HIF-PHI may be effective in improving anemia, the effect on improving renal function may be limited. Particularly in Stage 5, the responsiveness to the pleiotropic effects of HIF-PHIs, such as anti-inflammatory effects and fibrosis inhibition, may be further diminished compared with Stage 4. A more detailed analysis is needed to determine whether the effect of HIF-PHI varies according to the CKD stage.

The proportion of patients on daprodustat was the highest among the HIF-PHIs evaluated in this study. The results of this study strongly reflect the effects of daprodustat. PHIs have three domains, including PHD-1, −2, and −3. HIF-PHIs inhibit the differential specificity of these PHD isoforms [35]. Roxadustat inhibits PHD-1, −2, and −3, whereas daprodustat only inhibits PHD-1 and −3. It is not well established which PHD isoforms are inhibited by other HIF-PHIs, and the effects of these differences on clinical efficacy remain uncertain. Further studies are needed to determine whether the eGFR retention effect of HIF-PHIs results in a class effect or specific HIF-PHIs.

In summary, we demonstrated that HIF-PHI maintains eGFRcre for a year more than ESAs, suggesting that HIF-PHIs unique effects, other than hemoglobin improvement, are relevant. The results also indicated that the effect may vary depending on the CKD stage.

### Limitation

This study has some limitations. First, the doses of HIF-PHIs and ESAs were inconsistent in this study. Second, we were only able to assess eGFRcre in the short term after the initiation of HIF-PHI. Third, the proportion of HIF-PHIs incorporated was biased. Fourth, only Japanese patients were included in the analysis. Lastly, we could not examine the drugs added after the initiation of HIF-PHI. The added drugs are listed (Table S2); however, the timing of initiation varied from case to case, making it challenging to evaluate them in this study. Particular attention should be paid to SGLT2-i and ARNI because of their renoprotective effects. To minimize the influence of these drugs, only cases in which these drugs were not used were analyzed, and the results were similar to those of the present study (Table S3). Additionally, no other anemia markers, such as hepcidin, were assessed in this study. In addition, although the HIF-PHI and ESA groups were managed under similar clinical conditions in our tertiary care setting, selection bias due to differential loss to follow-up cannot be entirely excluded. Furthermore, as this study used prescription data exclusively from our hospital, medications prescribed at other institutions were not captured. We also reviewed the prescription records from our institution’s electronic medical system as comprehensively as possible; however, the possibility of unrecorded prescriptions cannot be completely ruled out. Therefore, residual confounding due to incomplete medication information and potential prior exposure to HIF-PHIs or ESAs at other hospitals cannot be entirely ruled out. Moreover, patients who received blood transfusions during the study were excluded from the primary analysis due to concerns about confounding renal function outcomes. Although this exclusion may have led to selection bias, sensitivity analyses including these patients yielded similar results (Table S5), supporting the robustness of the primary findings.

## Conclusions

HIF-PHIs preserved eGFRcre for 1 year, suggesting that its effect was superior to that of ESAs. Furthermore, the results indicated that the effect may vary according to the CKD stage.

## Electronic supplementary material

Below is the link to the electronic supplementary material.

Supplementary material 1

## Data Availability

The deidentified data of participants will not be shared.

## List of abbreviations

ACE-I: Angiotensin-converting enzyme inhibitor
ARB: Angiotensin receptor blocker
ARNI: Angiotensin receptor neprilysin inhibitor
CKD: Chronic kidney disease
eGFR: estimated glomerular filtration rate
ESAs: Erythropoiesis-stimulating agents
ESKD: End-stage kidney disease
EPO: Erythropoietin
HIF-PHIs: Hypoxia-inducible factor prolyl hydroxylase inhibitors
MRA: Mineralocorticoid receptor antagonist
PHD: Prolyl hydroxylase
RRT: Renal replacement therapy
SGLT2-I: Sodium-glucose co-transporter 2 inhibitors
TIBC: Total iron binding capacity
TSAT: Transferrin saturation
