# Supplementary material for: Preservation of eGFRcre for 1 year with HIF-PHI in non-dialysis patients: a retrospective observational cohort study
Source: J Pharm Health Care Sci. 2025 Dec 20;12:10. doi: 10.1186/s40780-025-00527-1 (PMC12838504; doi:10.1186/s40780-025-00527-1)
Supplement: Supplementary file 1 — Supplementary material 1 [file 40780_2025_527_MOESM1_ESM.docx]

# **Title:** **Preservation of eGFRcre for 1 Year with HIF-PHI in Non-Dialysis Patients: A Retrospective Observational Cohort Study**

**Journal name**: *Journal of Pharmaceutical Health Care and Sciences*

**author names**

Tomohiro Aigami^1^, Tomoyuki Ishigo^1^, Mai Miyao^1^, Masatoshi Nonoyama^1^, Tomohisa Yamashita^2^, Masayuki Koyama^3,4^, Satoshi Fujii^1^, Toshiyuki Yano^3^, Masato Furuhashi^3^, Masahide Fukudo^1*^, Takaki Toda^5^

^1^ Department of Pharmacy, Sapporo Medical University Hospital, Sapporo, Japan

^2^ Department of Nephrology and Dialysis Therapy, Sapporo Central Hospital, Sapporo, Japan

^3^ Department of Cardiovascular, Renal, and Metabolic Medicine, Sapporo Medical University School of Medicine, Sapporo, Japan

^4^ Department of Public Health, Sapporo Medical University School of Medicine, Sapporo, Japan.

^5^ Department of Clinical Pharmacology, Faculty of Pharmaceutical Sciences, Hokkaido University of Science, Sapporo, Japan

**Corresponding author**

Masahide Fukudo, Ph.D.

E-mail address: [fukudom@sapmed.ac.jp](mailto:fukudom@sapmed.ac.jp)

**Supplementary Material**


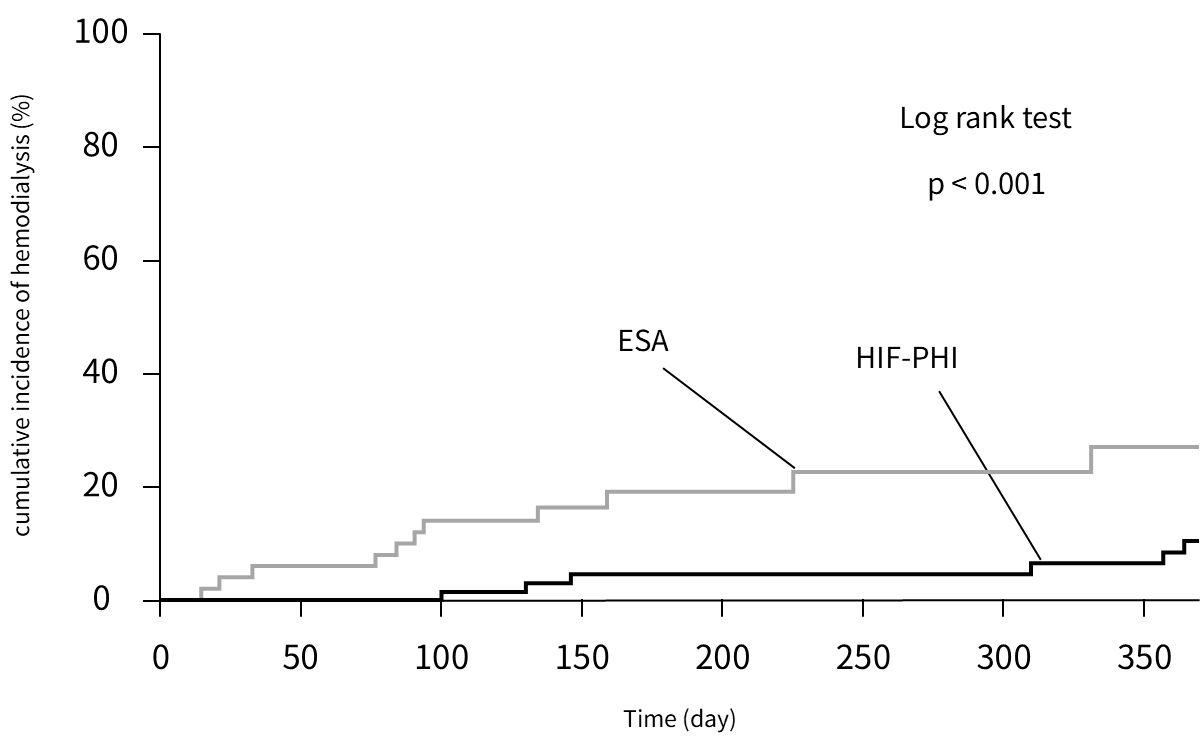


Fig. S1

The cumulative incidence of hemodialysis after HIF-PHI or ESA initiation.

Abbreviations: HIF-PHI, Hypoxia-inducible factor prolyl hydroxylase inhibitor; ESA, erythropoiesis-stimulating agent


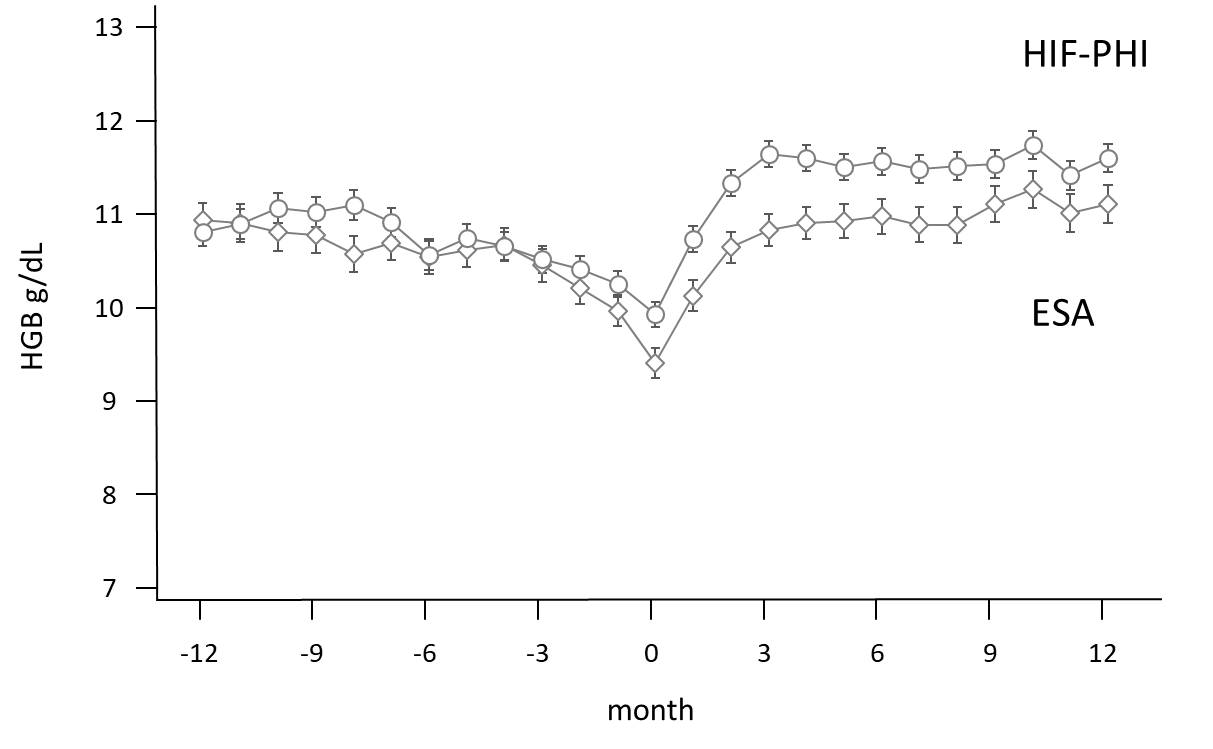


Fig. S2

The hemoglobin trend in HIF-PH and ESA.

Abbreviations: HGB, hemoglobin; HIF-PHI, Hypoxia-inducible factor prolyl hydroxylase inhibitor; ESA, erythropoiesis-stimulating agent


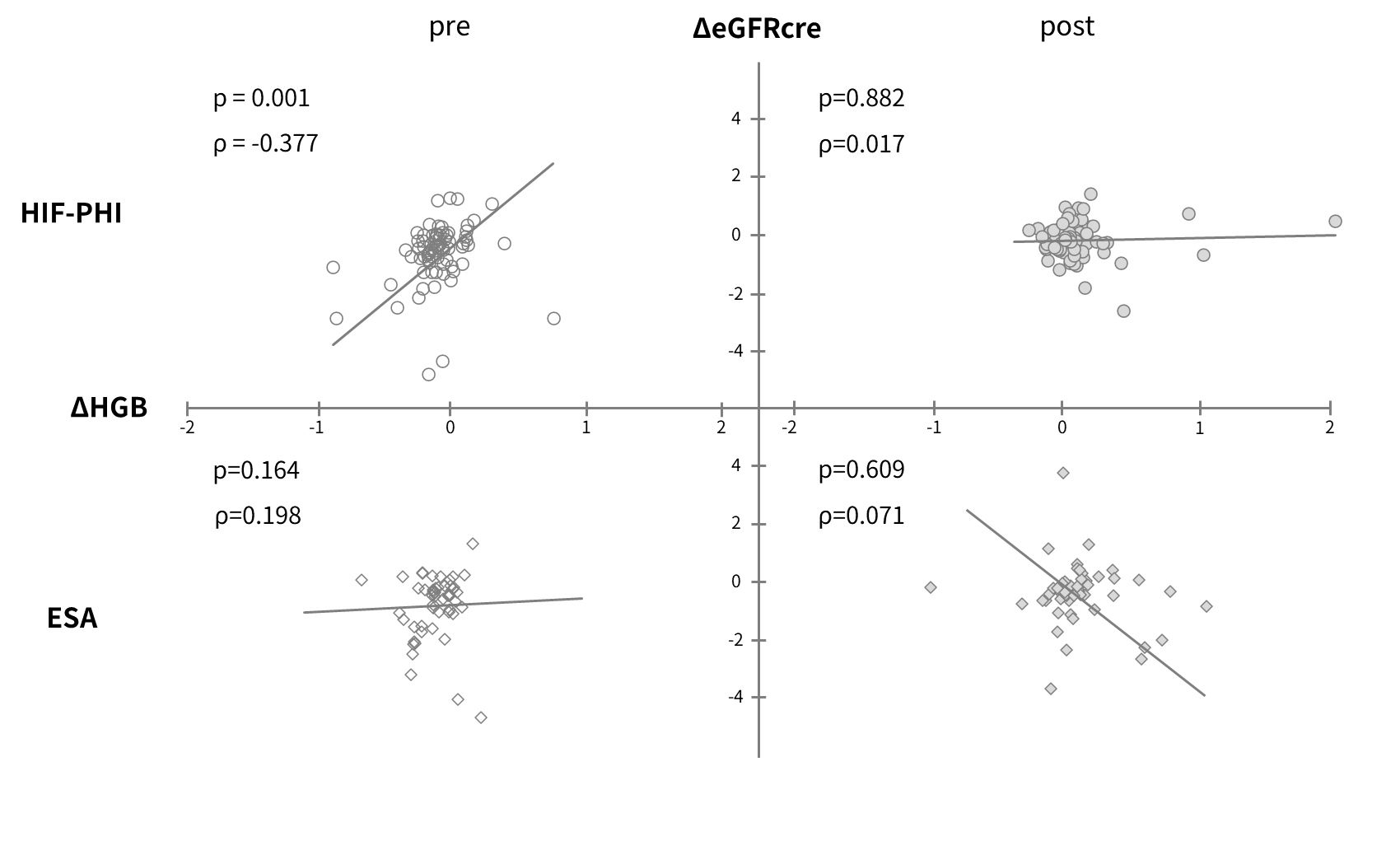


Fig. S3

The correlations between eGFRcre slope and hemoglobin trend.

Abbreviations: HIF-PHI, Hypoxia-inducible factor prolyl hydroxylase inhibitor; ESA, erythropoiesis-stimulating agent


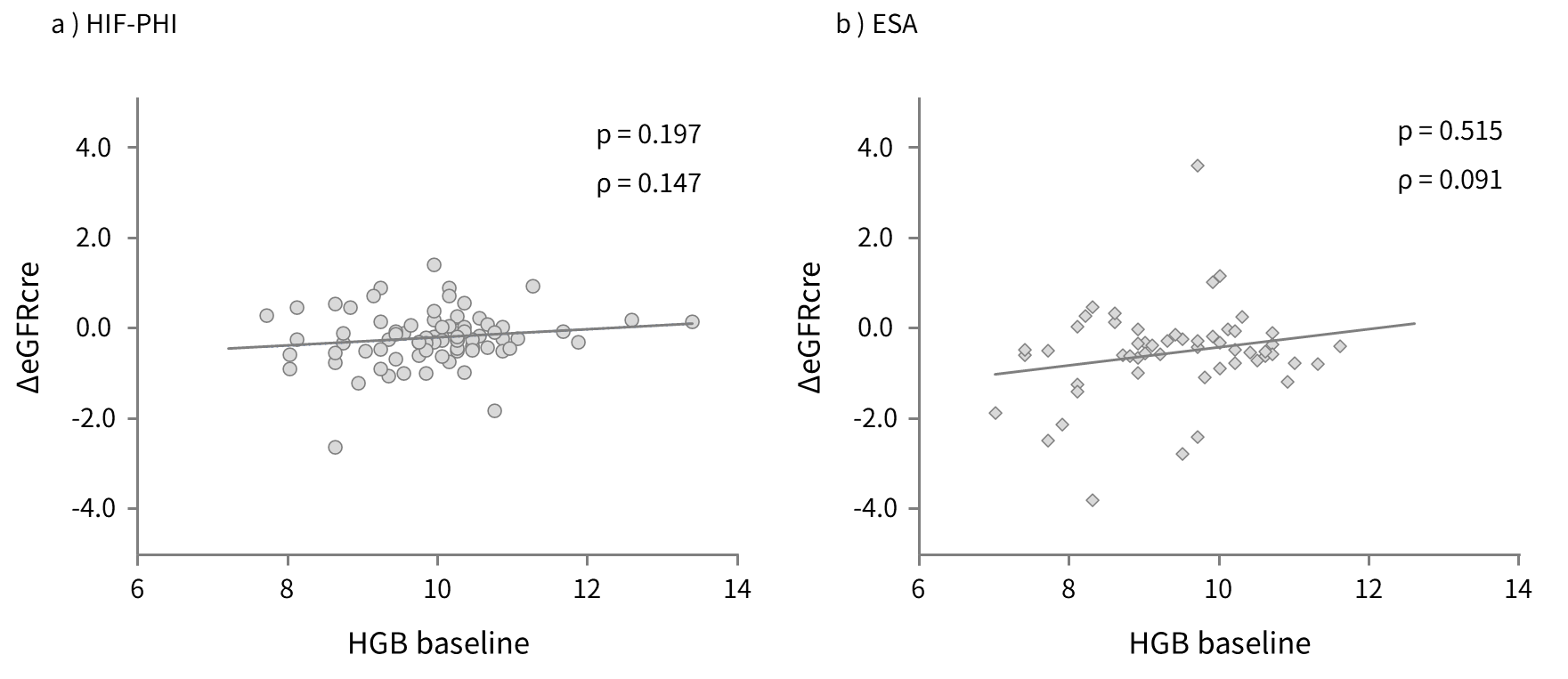


Fig. S4

The correlations between eGFRcre slope and baseline hemoglobin levels.

Abbreviations: HIF-PHI, Hypoxia-inducible factor prolyl hydroxylase inhibitor; ESA, erythropoiesis-stimulating agent


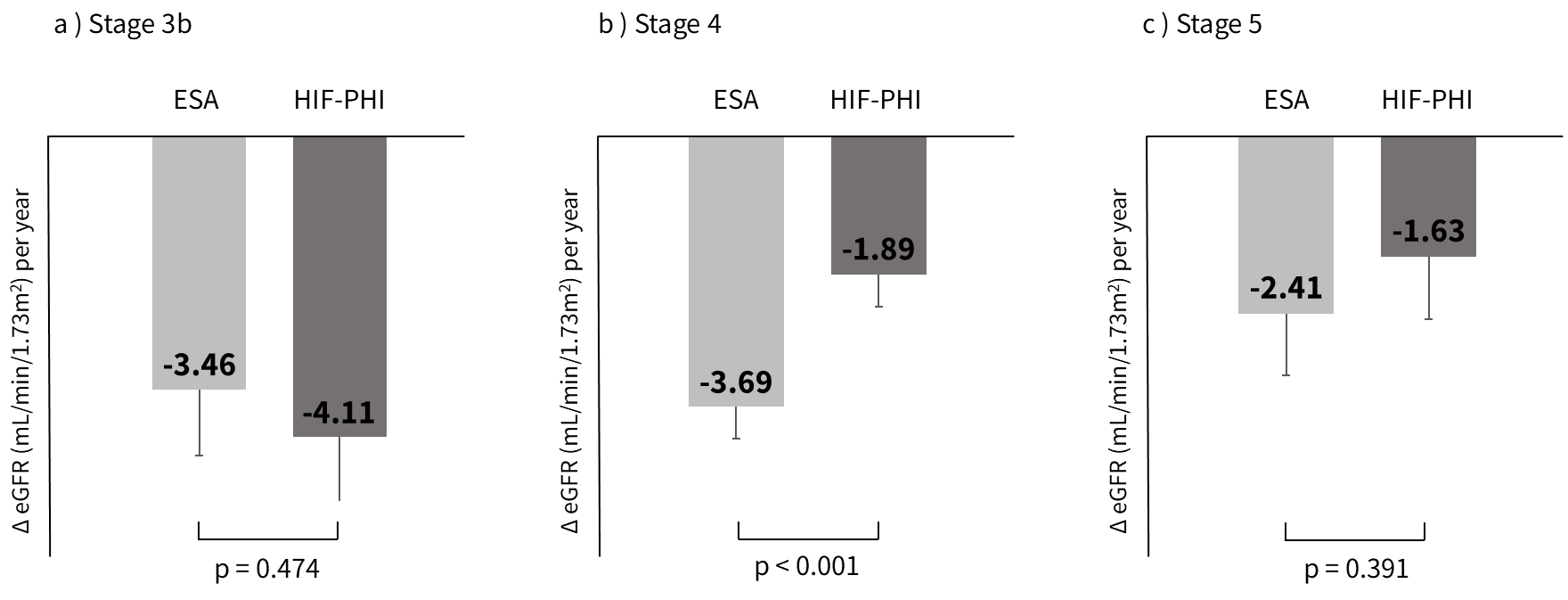


Fig. S5

The post-treatment eGFRcre slope of the HIF-PHI and ESA groups based on the CKD stage.

Abbreviations: HIF-PHI, Hypoxia-inducible factor prolyl hydroxylase inhibitor; ESA, erythropoiesis-stimulating agent

**Table S1 The proportions of HIF-PHI species and ESA species**

|  | HIF-PHI  n= 79 |  |  | ESA  n= 55 |
| --- | --- | --- | --- | --- |
| Species |  |  | **Species** |  |
| Roxadustat n (%) | 12 (15.2) |  | darbepoetin alpha n (%) | 14 (25.5) |
| Vadadustat n (%) | 6 (7.6) |  | epoetin beta pegol n (%) | 41 (74.5) |
| Daprodustat n (%) | 53 (67.1) |  |  |  |
| Enarodustat n (%) | 5 (6.3) |  |  |  |
| Molidustat n (%) | 3 (3.8) |  |  |  |

Values are presented as numbers (with percentages).

**Table S2 Add on medications after HIF-PHI or ESA initiation**

|  | ALL  n= 134 | HIF-PHI  n= 79 | ESA  n= 55 | p-value |
| --- | --- | --- | --- | --- |
| Add on medications |  |  |  |  |
| NSAIDs n (%) | 2 (1.5) | 2 (2.6) | 0 (0) | 0.232 |
| ARB/ACE-i n (%) | 7 (5.2) | 4 (5.1) | 3 (5.5) | 0.920 |
| ARNI n (%) | 21 (15.78) | 17 (21.5) | 4 (7.3) | 0.026 |
| MRA n (%) | 8 (6.0) | 3 (3.8) | 5 (9.1) | 0.203 |
| Loop diuretic n (%) | 16 (11.9) | 10 (12.7) | 6 (10.9) | 0.759 |
| SGLT2-i n (%) | 17 (12.7) | 15 (19.0) | 2 (3.6) | 0.009 |
| iron supplementation n (%) | 25 (18.8) | 14 (18.0) | 11 (20) | 0.766 |

Values are presented as numbers (with percentages). p <0.05 was considered statistically significant. Abbreviations: NSAIDs, non-steroid anti-inflammatory drugs; ARB, angiotensin receptor blocker; ACE-i, angiotensin-converting enzyme inhibits; ARNI, angiotensin receptor neprilysin inhibitor; MRA, mineralocorticoid receptor antagonist; SGLT2-i, sodium-glucose co-transporter 2 inhibitors.

**Table S3 Multivariable analysis excluding patients prescribed ARNI or SGLT-2i**

|  | **Model 1** | | |  | **Model 2** | | |
| --- | --- | --- | --- | --- | --- | --- | --- |
| **Fix effect** | **Estimate (SE)** | **95 % CI** | **p-value** |  | **Estimate (SE)** | **95 % CI** | **p-value** |
| **Intercept** | 27.93 (1.14) | 25.69 to 30.18 | < 0.001 |  | 25.12 (4.46) | 16.26 to 33.98 | < 0.001 |
| **Drug (ESA)** | 0.548 (1.02) | -1.47 to 2.57 | 0.592 |  | -0.184 (0.62) | -1.42 to 1.05 | 0.768 |
| **Time month** | -0.378 (0.04) | -0.46 to -0.30 | < 0.001 |  | -0.373 (0.04) | -0.45 to -0.30 | < 0.001 |
| **Drug*time** | -0.106 (0.04) | -0.18 to -0.03 | 0.009 |  | -0.104 (0.04) | -0.18 to -0.03 | 0.008 |
| **Age** |  |  |  |  | 0.060 (0.05) | -0.04 to 0.16 | 0.230 |
| **Sex (female)** |  |  |  |  | 0.012 (0.63) | -1.24 to 1.27 | 0.985 |
| **BMI** |  |  |  |  | -0.054 (0.13) | -0.31 to 0.20 | 0.672 |
| **CKD stage** |  |  |  |  |  |  |  |
| **3b** |  |  |  |  | 10.09 (1.00) | 8.12 to 12.1 | < 0.001 |
| **4** |  |  |  |  | 1.44 (0.85) | -0.25 to 3.14 | 0.095 |

p <0.05 was considered statistically significant. Abbreviations: SE, standard error; ESA, erythropoiesis-stimulating agents; BMI, body mass index; CKD, chronic kidney disease.

**Table S4 Multivariable analysis incorporating prior HF as a covariate.**

|  | **Model 1** | | |  |
| --- | --- | --- | --- | --- |
| **Fix effect** | **Estimate (SE)** | **95 % CI** | **p-value** |  |
| **Intercept** | 23.40 (3.75) | 15.97 to 30.82 | < 0.001 |  |
| **Drug (ESA)** | -1.05 (0.53) | -2.10 to -0.01 | 0.049 |  |
| **Time month** | -0.18 (0.05) | -0.27 to -0.08 | < 0.001 |  |
| **Drug*time** | -0.10 (0.05) | -0.19 to -0.01 | 0.048 |  |
| **Age** | 0.05 (0.04) | -0.03 to 0.13 | 0.238 |  |
| **Sex (female)** | -0.65 (0.52) | -1.68 to 0.38 | 0.214 |  |
| **BMI** | -0.07 (0.11) | -0.28 to 0.14 | 0.526 |  |
| **CKD stage** |  |  |  |  |
| **3b** | 10.89 (0.82) | 9.27 to 12.51 | < 0.001 |  |
| **4** | 0.24 (0.68) | -1.12 to 1.59 | 0.731 |  |
| **HF** | -0.97 (0.59) | -2.14 to 0.20 | 0.104 |  |

p <0.05 was considered statistically significant. Abbreviations: SE, standard error; ESA, erythropoiesis-stimulating agents; BMI, body mass index; CKD, chronic kidney disease; HF, Heart failure.

**Table S5 Multivariable analysis incorporating history of blood transfusions during study.**

|  | **Model 1** | | |  |
| --- | --- | --- | --- | --- |
| **Fix effect** | **Estimate (SE)** | **95 % CI** | **p-value** |  |
| **Intercept** | 21.88 (3.55) | 14.88 to 28.89 | < 0.001 |  |
| **Drug (ESA)** | -0.76 (0.49) | -1.73 to 0.20 | 0.120 |  |
| **Time month** | -0.17 (0.05) | -0.26 to -0.08 | < 0.001 |  |
| **Drug*time** | -0.10 (0.05) | -0.19 to -0.01 | 0.039 |  |
| **Age** | 0.06 (0.04) | -0.01 to 0.14 | 0.109 |  |
| **Sex (female)** | -0.82 (0.49) | -1.79 to 0.15 | 0.098 |  |
| **BMI** | -0.07 (0.04) | -0.27 to 0.14 | 0.515 |  |
| **CKD stage** |  |  |  |  |
| **3b** | 10.99 (0.78) | 9.46 to 12.52 | < 0.001 |  |
| **4** | 0.06 (0.64) | -1.21 to 1.34 | 0.920 |  |

p <0.05 was considered statistically significant. Abbreviations: SE, standard error; ESA, erythropoiesis-stimulating agents; BMI, body mass index; CKD, chronic kidney disease.
